# The Reality of Lung Cancer Paradox: The Impact of Body Mass Index on Long-Term Survival of Resected Lung Cancer. A French Nationwide Analysis from the Epithor Database

**DOI:** 10.3390/cancers13184574

**Published:** 2021-09-12

**Authors:** Marco Alifano, Elisa Daffré, Antonio Iannelli, Laurent Brouchet, Pierre Emmanuel Falcoz, Françoise Le Pimpec Barthes, Alain Bernard, Pierre Benoit Pages, Pascal Alexandre Thomas, Marcel Dahan, Raphael Porcher

**Affiliations:** 1 Thoracic Surgery Department, Cochin Hospital, University of Paris, 75014 Paris, France; elisa.daffre@aphp.fr; 2 Digestive Surgery Unit, Archet 2 Hospital, University Hospital of Nice, 06108 Nice, France; iannelli.a@chu-nice.fr; 3 Thoracic Surgery Department, Hôpital Larrey, CHU Toulouse, 31000 Toulouse, France; l.brouchet@sfctcv.org (L.B.); m.dahan@sfctcv.org (M.D.); 4 Thoracic Surgery Department, Nouvel Hôpital Civil de Strasbourg, University of Strasbourg, 67000 Strasbourg, France; p.falcoz@sfctcv.org; 5 Thoracic Surgery Department, Hôpital Européen Georges Pompidou, University of Paris, 75015 Paris, France; francoise.lepimpec-barthes@aphp.fr; 6 Thoracic Surgery Department, Dijon University Hospital, 21000 Dijon, France; a.bernard@sfctcv.org (A.B.); pierrebenoit.pages@chu-dijon.fr (P.B.P.); 7 Thoracic Surgery Department, Hopital-Nord-APHM, Aix-Marseille University, 13005 Marseille, France; pascalalexandre.thomas@ap-hm.fr; 8 Centre of Research Epidemiology and Statistics (CRESS), University of Paris, INSERM U1153, 75014 Paris, France; raphael.porcher@aphp.fr

**Keywords:** lung cancer, BMI, obesity

## Abstract

**Simple Summary:**

It is commonly believed that obesity increases the risk of cancers and lowers the possibility of cure of patients with proven cancers. In recent years, this traditional view has been challenged by the hypothesis of an ‘obesity paradox’, which refers to a better prognosis in obese patients with some specific cancers, compared to normal/underweight patients. In this study, we assessed, in a nationwide dataset, the prognostic role of preoperative BMI on postoperative outcomes in patients undergoing curative lung resection for non-small-cell lung cancer (NSCLC) and found that BMI is a strong and independent predictor of long-term survival.

**Abstract:**

Obesity could have a protective effect in patients with lung cancer. We assessed the prognostic role of preoperative BMI on survival in patients who underwent lung resection for NSCLC. A total of 54,631 consecutive patients with resectable lung cancer within a 15-year period were extracted from Epithor (the French Society of Thoracic and Cardiovascular Surgery database). Patient subgroups were defined according to body mass index (BMI): underweight (BMI < 18.5 kg/m^2^), normal weight (18.5 ≤ BMI < 25 kg/m^2^), overweight (25 ≤ BMI < 30 kg/m^2^), and obese (BMI ≥ 30 kg/m^2^). Underweight was associated with lower survival (unadjusted HRs 1.24 (1.16–1.33)) compared to normal weight, whereas overweight and obesity were associated with improved survival (0.95 (0.92–0.98) and 0.88 (0.84–0.92), respectively). The impact of BMI was confirmed when stratifying for sex or Charlson comorbidities index (CCI). Among patients with obesity, a higher BMI was associated with improved survival. After adjusting for period of study, age, sex, WHO performance status, CCI, side of tumor, extent of resection, histologic type, and stage of disease, the HRs for underweight, overweight, and obesity were 1.51 (1.41–1.63), 0.84 (0.81–0.87), and 0.80 (0.76–0.84), respectively. BMI is a strong and independent predictor of survival in patients undergoing surgery for NSCLC.

## 1. Introduction

During the last few decades, the prevalence of obesity has increased dramatically and become a worldwide health problem [1]. According to the World Health Organization (WHO), the normal body mass index (BMI) range is between 18.5 and 25 kg/m^2^ [1]. Many studies suggest a strong correlation between obesity and many metabolic disturbances, including hypertension, diabetes, hyperlipidemia, and non-alcoholic fatty liver disease [2,3]. Recently, a study using data from 386,101 UK Biobank participants aged from 37 to 73 years with a mean follow-up of 8.8 years, evaluated cancer incidence at 22 sites: compared to participants with normal weight and waist circumference, overweight or obese men with increased central adiposity had a higher risk of cancer incidence at three sites, namely stomach, kidney, and colorectal. Women had a higher risk of cancer incidence at endometrial, uterine, kidney, and breast sites, but only endometrial cancer mortality was significantly associated with being overweight and centrally obese [3]. Biological mechanisms behind the major role of obesity in chronic inflammation and carcinogenesis have been extensively assessed [4,5,6,7]. On the other hand, a population-based cohort study on BMI and the risk of 22 specific cancers in 5.24 million UK adults, showed that BMI was associated with 17 of 22 cancers, but the effect varied substantially by cancer type; each 5 kg/m² increase in BMI was linearly associated with cancers of the uterus, gallbladder, kidney, cervix, thyroid, and leukemia [8]. Less strongly, BMI was positively associated with liver, colon, ovarian, and postmenopausal breast cancers. However, an inverse association was found with prostate and premenopausal breast cancer risk, both overall, and in never-smokers. By contrast, for lung and oral cavity cancer, no association was observed in never-smokers, but an inverse association was found between BMI and occurrence of these cancers in current and ex-smokers, suggesting a different behavior for these malignancies [8]: for lung cancer, the expression “lung cancer paradox” was suggested [9].

The impact of BMI on short and long-term outcomes of different cancers has been assessed less extensively than incidence, but an increasing research interest exists. A 2021 meta-analysis, including 45 studies encompassing more than 600,000 patients, showed that obesity was associated with increased odds of overall and cancer-specific mortality in colorectal carcinoma compared to normal weight [10]. However, individuals with underweight BMI also had increased odds of cancer-specific mortality compared to normal BMI and this was not significantly different from obesity. A recent retrospective cohort study using data from the Veterans Health Administration Corporate Data Warehouse on patients with esophageal, cardia, and non-cardia gastric adenocarcinomas showed that a premorbid or at-diagnosis BMI of 18 or 20 bears a worse prognosis than a normal or overweight BMI. Interestingly, in locally advanced cancer, no association between at-diagnosis BMI and mortality could be demonstrated after adjusting for weight loss. These data indicate that for early-stage upper-GI cancers, obesity has a protective effect, the obesity paradox, which does not hold for locally advanced cancers, for which only weight loss (not BMI) impacts survival [11].

In lung cancer, institutional series showed that BMI > 25 kg/m^2^ was associated with improved long-term survival after surgical resection; however, confirmation by large-scale studies is required [12,13]. In a recent retrospective analysis of the Japanese Joint Committee of Lung Cancer Registry, univariate analysis showed a negative association between underweight and long-term survival and a non-significantly lower risk of BMI > 25 kg/m^2^ (without differentiating overweight from obesity); however, this difference became significant after adjusting for relevant covariates [14].

Thus, it seems necessary to use a large-scale setting to assess the impact of four major BMI categories (underweight, normal weight, overweight, obesity) on the postoperative survival of resected lung cancer. In this study, we assessed the prognostic role of preoperative BMI on postoperative outcomes in patients who underwent curative lung resection for non-small-cell lung cancer (NSCLC) whose data were prospectively collected in Epithor, the official database of the French Society of Thoracic and Cardiovascular Surgery (FSTCVS).

## 2. Materials and Methods

The Institutional Review Board of the FSTCVS approved the study (CERC-SFCTCV-22 February 2021-Num02_ImpactIMC-Cancer). Patient consent was obtained for entry into the database, and patients were aware that these data would be used for research purposes.

### 2.1. Epithor, the French National Database of General Thoracic Surgery

Epithor was created in 2002 as a voluntary and free initiative of general thoracic surgeons in France. It is the official database of the FSTCVS. Currently, 111 centers contribute to the database, which includes most surgical procedures performed in French thoracic surgical departments. The list of centers participating to Epithor is shown in Supplementary materials.

Data-quality monitoring is financially supported by the French National Cancer Institute (INCA). As a methodologically correct tool to assess surgical practices, Epithor is endorsed by the French National High Authority for Health (HAS), the governmental agency in charge of improving quality of care and guaranteeing the adequateness of the whole health care system in providing state-of-the-art care.

Previous reports have focused on the technical characteristics of Epithor [15,16]. In particular, the use of hierarchic pull-down menus and the absence of free text space facilitates completeness and accuracy of the data. Routine utilities for data consistency and alerting against aberrant or contradictory values are incorporated in the software. Overall, 52 variables can be collected per patient, covering information about the patients’ characteristics, associated illness, pulmonary function, surgical procedures, cancer staging, and postoperative outcome.

Epithor includes functions allowing participating surgeons to benchmark their activity against the national picture, by comparing the local database with the national one for completeness. This comparison is expressed through a quality score ranging from 0 to 100%. Since 2010, the accuracy of data collection is checked in regular external onsite audits.

### 2.2. Patient Population

**Figure 1:**
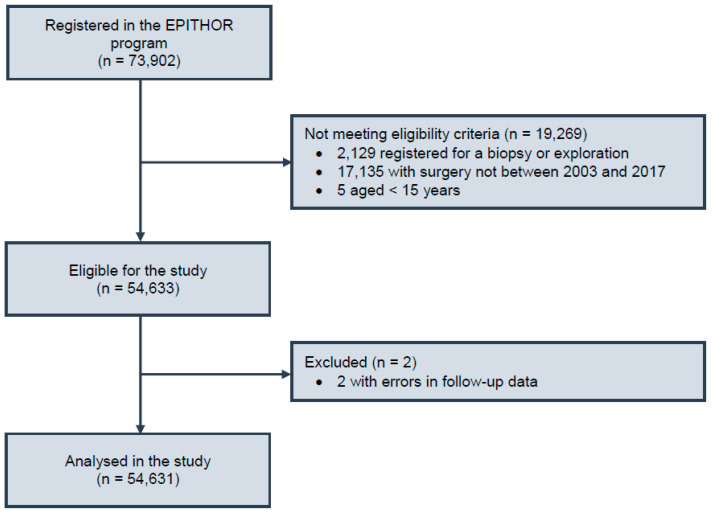
Study flow chart.

Epithor includes 73,902 patients with a definitive diagnosis of lung cancer (Figure 1). To account for the progressive implementation of the Epithor project by different surgical centers in 2002 and to include at least 3 years of follow-up for all the patients, we extracted data of patients aged 15 or over, who were operated on with curative intent between 1 January 2003, and 31 December 2017. After excluding two patients with obvious errors in follow-up dates, a study sample of 54,631 patients having undergone lung resection for primary lung cancer was selected for further analysis (Figure 1). From this population, patients were divided into four groups based on their BMI: underweight (BMI < 18.5 kg/m^2^), normal weight (18.5 ≤ BMI < 25 kg/m^2^), overweight (25 ≤ BMI < 30 kg/m^2^), and obese (BMI ≥ 30 kg/m^2^).

### 2.3. Retrieved Clinical Variables

Baseline demographics, comorbidities, procedure, and outcome were recorded. Patient-related variables included age, sex, weight, height, BMI, American Society of Anesthesia score, WHO performance status, and comorbid diseases. Based on the information on comorbid diseases, the Charlson comorbidity index (CCI) was calculated for each case [17]. Surgery-related variables included: side of the procedure, extent of exeresis, pathological staging using the International Association for the Study of Lung Cancer classification (early I–II, locally advanced III, and metastatic IV), and histologic type. Date of surgery but not date of initial diagnosis is available in Epithor. Of note, according to INCA guidelines, surgery is performed within 30 days from establishment of diagnosis [18].

### 2.4. Outcome Definition

The primary endpoint was overall survival of up to 5 years. Thirty and 90-day mortality were calculated. The vital status of patients was checked by the National Institute for Statistics and Economy website.

### 2.5. Statistical Analysis

Descriptive data were expressed as frequency and percentage for qualitative variables, and continuous variables, as mean and standard deviation. Association of BMI with clinical variables was assessed using ANOVA, Kruskal–Wallis, and χ2 tests. Survival curves were obtained by the Kaplan–Meier estimator. Prognostic analyses were performed using Cox proportional hazards models, with censoring at 5 years to allow adjusting for the period which included the years 2015–2017. The shape of the hazard ratio for BMI and CCI was estimated using flexible splines, then both variables were categorized for further analyses, allowing the display of survival curves by category. The proportional hazards assumption was evaluated by examination of Schoenfeld residuals and Grambsch–Therneau’s lack-of-fit test. Three multivariable models were built to adjust for an increasing number of other factors. All those adjustment factors were determined a priori based on clinical significance as potential prognostic factors, and the three sets were constructed so that models would be fitted on datasets with increased proportion of missing data (from <1% to about 30%). Analyses stratified by sex were carried out, and the association of BMI categories, unadjusted and adjusted for the three sets of covariates, were compared between females and males using an interaction test. Preplanned exploratory analyses included stratified analyses by CCI category and refined BMI categories among participants with BMI ≥ 30 kg/m^2^. A post-hoc analysis stratified by tumor histology was subsequently added.

Missing data were handled through multiple imputations by chained equations using outcomes as well as all confounders mentioned above in the imputation model. Missing baseline data were handled through multiple imputations by chained equations, using the baseline hazard of death and all variables used in the Cox models in the imputation model [19]. Ten independent imputed data sets were generated and analyzed separately. Estimates were then pooled using Rubin’s rules. All analyses were carried out using the R statistical software version 3.6.3 (R Foundation for Statistical Computing, Vienna, Austria, 2020).

## 3. Results

The study included 54,631 patients who had undergone lung resection for primary lung cancer. The patient population is described in Table 1. Mean age was 64.1 (SD 10.1) years, 30.1% were women and 69.9% men. Median CCI was 4.4 (interquartile range, IQR 3–5); Performance Status (PS) were 0, 1, and 2–4 in 45.4%, 45.1%, and 9.4% of patients, respectively. Adenocarcinoma and squamous-cell carcinoma represented the more frequent histologic types, accounting for 59.0% and 27.7% of cases, respectively. Stage I disease accounted for 53.8% of cases; stages II and III accounted for 18.6% and 21.3% of patients, respectively, whereas only a minority of patients had oligometastatic disease (5.9%). Intervention was right-sided in 57.8% of patients and left-sided in 42.2% of cases, and 10.1% of patients had pneumonectomy. Mean BMI was 25.3 (SD 4.6), and 2468 patients (4.5%) were underweight, 25,482 (46.6%) were normal weight, 18,765 (34.3%) were overweight, and 7877 (14.4%) were obese. Among patients with obesity, 6170 (78.3%) had a BMI between 30 and 34.9 kg/m^2^, 1342 (17.0%) between 35 and 39.9 kg/m^2^, and 365 (4.7%) >40 kg/m^2^.

### 3.1. Correlations of BMI

Underweight was more frequent in women and younger patients, whereas overweight and obesity were more frequent in men and older patients (Table 1). CCI was lower in underweight compared to normal-weight patients and higher in overweight and obese individuals, whereas PS was lower in normal-weight patients, higher in overweight, and, to a greater extent, in underweight and obese patients. Pneumonectomy was performed less frequently in obese and underweight patients than in normal or overweight individuals. In obese patients, there was a higher proportion of stage I disease, whereas in underweight patients, stage IV was more frequent.

### 3.2. Survival

Median follow-up was 5.2 years (IQR 2.3–9.5). For the whole population, 30 and 90-day mortality was 2.6% and 4.7%, respectively. Overall survival at 1, 3, and 5 years was 87.2%, 69.5%, 58.4%, respectively (Supplementary Table S1). Overall survival at specific time points in relation to main clinical and pathologic variables is shown in Supplementary Table S1.

**Figure 2:**
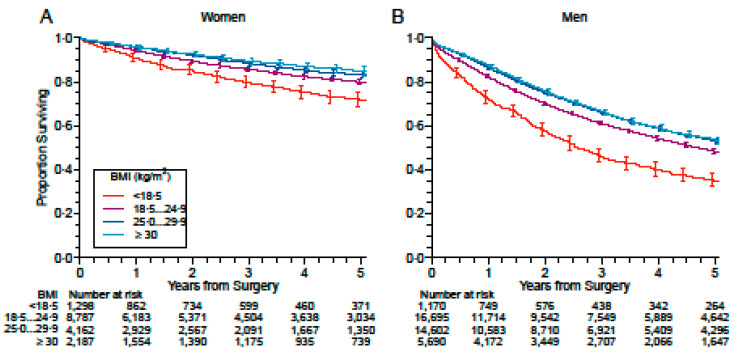
Kaplan–Meier survival curves of patients with resected lung cancer with respect to BMI. (**A**) Women. (**B**) Men.

**Figure 3:**
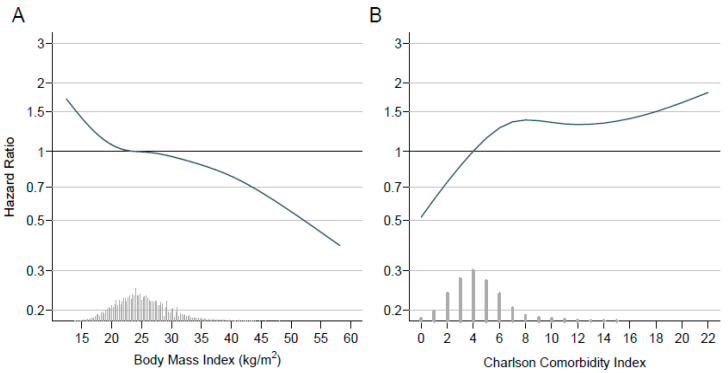
Unadjusted hazard ratio of body mass index (**A**) and Charlson comorbidity index (**B**). The hazard ratio (dark blue line) and 95% confidence interval (blue shaded region) are given for the whole range of observed values, relative to the mean BMI or CCI value. On the bottom of the plot, a histogram or bar chart shows the distribution of BMI or CCI values in the study.

Underweight was associated with lower survival than normal weight, whereas overweight and, to a greater extent, obesity was associated with improved survival (unadjusted HRs of 1.24 (95% CI 1.16–1.33), 0.95 (0.92–0.98), and 0.88 (0.84–0.92), respectively) (Figure 2, Table 2). Unadjusted HR at different non-categorical BMI values is shown in Figure 3A. For comparison, unadjusted HR at different CCI values is also shown in Figure 3B. The association with survival for the different BMI categories was confirmed and even straightened in the three models adjusted for potential confounders (Table 2); for instance, when adjusting for stage of disease, histology, side of tumor, extent of resection, PS, CCI, sex, and time frame of patient inclusion, HRs were 1.51 (1.41–1.63), 0.84 (0.81–0.87), and 0.80 (0.76–0.84) for underweight, overweight, and obesity, respectively. Results were similar when data without imputation were taken into account (Supplementary Table S2).

### 3.3. Stratification by Sex

As BMI was different between men and women, we stratified the analysis by sex, which confirmed the association of BMI with survival in each group. Unadjusted HRs for underweight, overweight, and obesity were 1.49 (1.29–1.71), 0.81 (0.72–0.90), and 0.72 (0.63–0.84) in women, and 1.51 (1.40–1.64), 0.85 (0.82–0.88), and 0.83 (0.79–0.88) in men, without significant difference between sexes (Table 3). All three adjustment models confirmed the impact on survival of different BMI categories after stratification by sex; for example, after adjusting for the stage of disease, histology, side of tumor, extent of resection, PS, CCI, and the time frame of patient inclusion, HRs were 1.38 (1.20–1.59), 0.82 (0.73–0.91), and 0.73 (0.63–0.84) in underweight, overweight, and obese women, and 1.55 (1.42–1.68), 0.84 (0.81–0.87), and 0.81 (0.77–0.86) in underweight, overweight, and obese men, respectively.

### 3.4. Subgroup Exploratory Analyses

**Figure 4:**
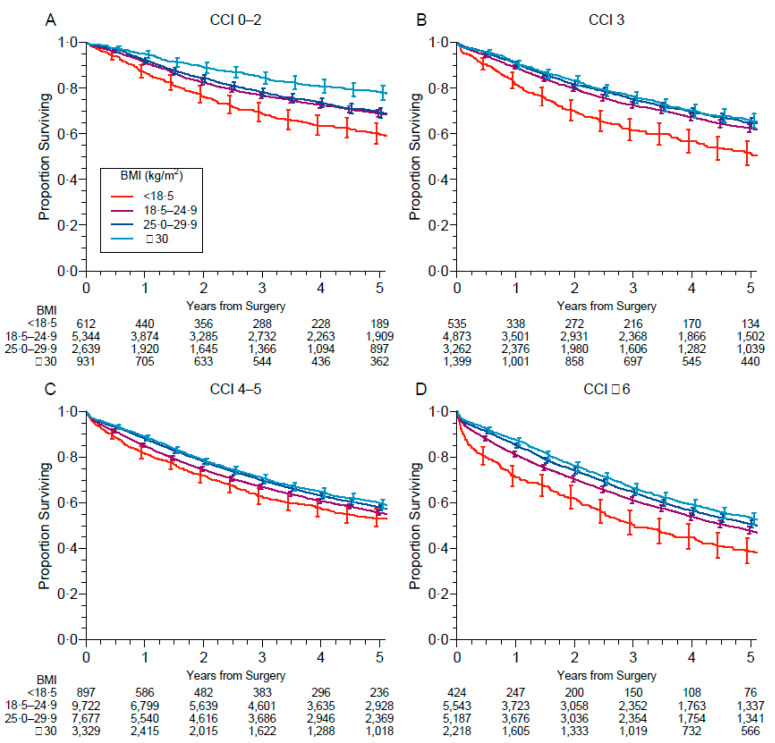
Survival according to BMI and Charlson comorbidity index (CCI) categories: 0–2 (**A**), 3 (**B**), 4–5 (**C**), >6 (**D**).

Figure 4 shows survival curves with respect to BMI categories in different CCI subgroups (0–2, 3, 4–5, and ≥6). Interestingly, the association of underweight with increased risk and the association of obesity with decreased risk seemed more pronounced not only in low-comorbidity patients but also in subjects with CCI ≥ 6.

**Figure 5:**
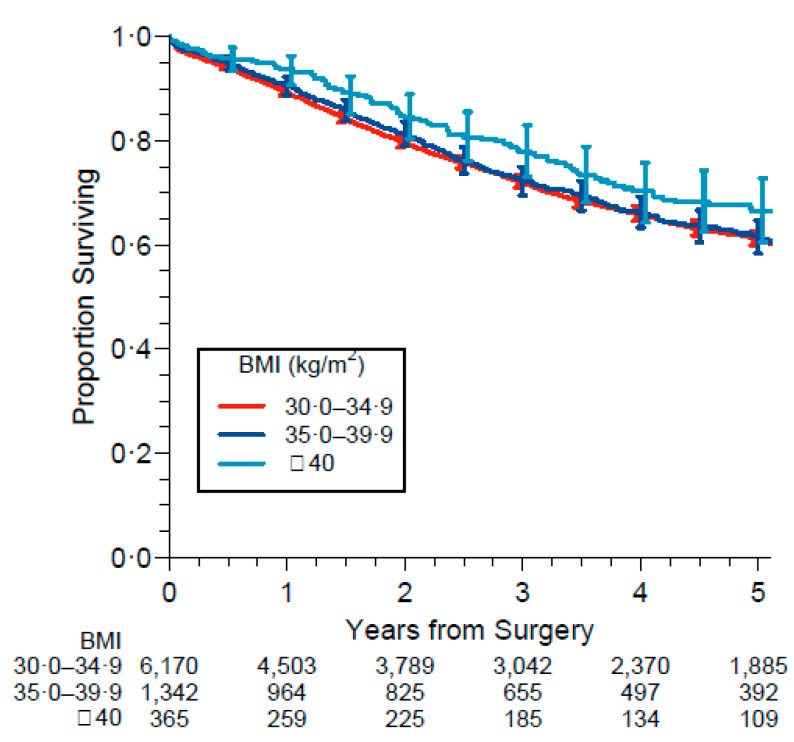
Survival according to obesity categories. Obesity classes are defined as class 1: BMI 30.0–34.9, class 2: BMI 35.0–39.9 and class 3: BMI ≥ 40.0.

Figure 5 shows survival according to obesity categories. Remarkably, the negative association of obesity with risk was stronger for higher BMIs, with a maximum protective effect in patients with BMI ≥ 40 kg/m^2^.

**Figure 6:**
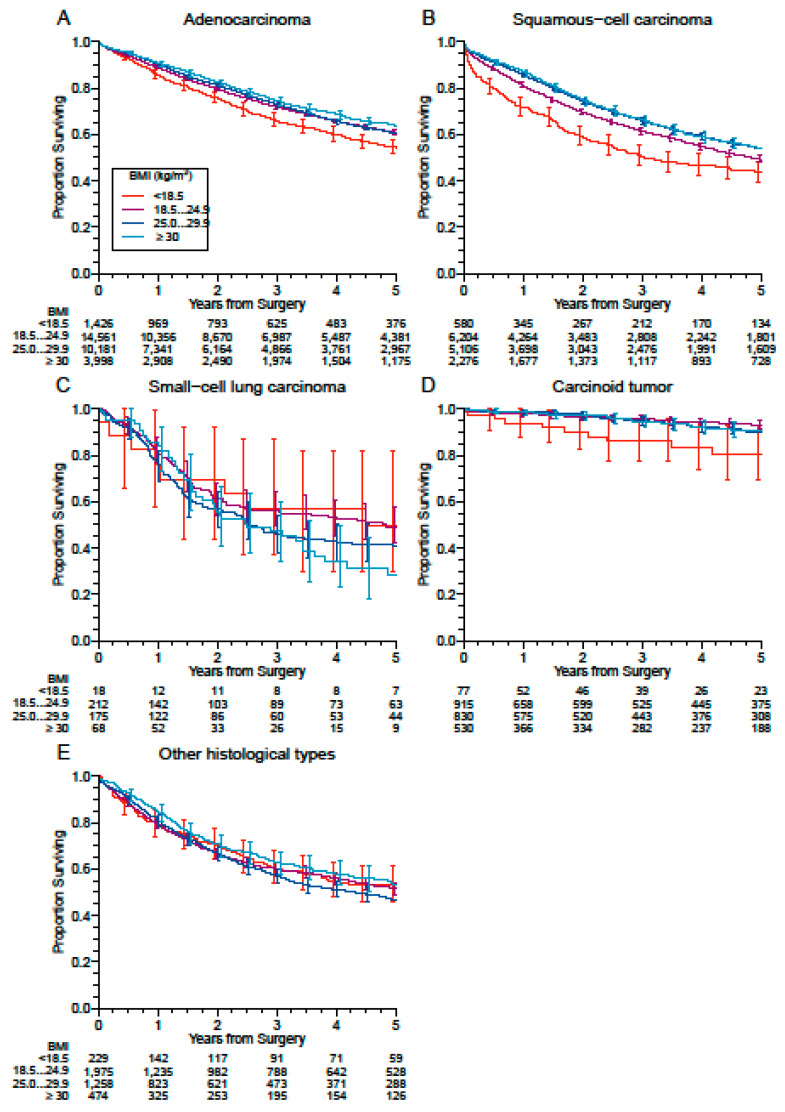
Survival according to obesity categories in different histologic types: (**A**) Adenocarcinoma, (**B**) Squamous-Cell Carcinoma; (**C**) Small-cell carcinoma; (**D**) Carcinoid tumors (typical and atypical); (**E**) Others. Obesity classes are defined as class 1: BMI 30.0–34.9, class 2: BMI 35.0–39.9 and class 3: BMI ≥ 40.0.

Figure 6 shows survival according to obesity categories in different histologic types. Remarkably, in both adenocarcinoma and squamous-cell carcinoma, as well as in carcinoid tumors the prognostic impact of different BMI categories remained evident.

## 4. Discussion

In the present study, we report evidence that BMI is a strong predictor of survival in patients undergoing radical surgery for NSCLC; underweight was associated with a worse prognosis, whereas overweight and, to a greater extent, obesity with a better prognosis. In particular, severe obesity is associated with an even better outcome. This impact of BMI on long-term survival mirrors the effect on perioperative outcome previously reported in resectable lung cancer [15].

We also show that the prognostic significance of obesity is independent from the “classical” prognostic factors of resectable lung cancer, namely pathologic stage, histologic type, extent of resection, sex, age, PS, or CCI [20,21], and the prognostic value of these factors is confirmed in our study. Stratification by sex also provided interesting results, as the impact of BMI was confirmed in a separate analysis of men and women and seemed slightly more pronounced in women.

In contrast to the evidence that obesity increases the risk of 13 different cancer types not including lung cancer [8], there is an ongoing debate on the impact of obesity on the long-term survival of cancer patients, with somewhat paradoxical results from some studies, a phenomenon called “the obesity paradox” [22,23]. On the other hand, the “lung cancer paradox” phenomenon would refer to the protective effect of higher BMI on both incidence, which is definitively established, and post-treatment outcome of lung cancer which requires definitive confirmation [9].

It has been stated that in cancers other than lung cancer, the observed obesity paradox could be explained by several methodological limitations including confounding factors, reverse causation, and collider stratification bias [24]. Ultimately, BMI is not a completely adequate measure of adiposity in cancer patients who often experience changes in body weight and body composition because of disease and treatments; however, pre-disease body weight is often not available [25]. It has also been found that lack of consideration for variability in the strength and directions of associations by age, sex, race/ethnicity, and cancer subtype could jeopardize results of studies in different cancer types aimed to definitively assess the question [26]. For instance, in locally advanced upper-GI cancer, only the at-diagnosis BMI is associated with mortality, and this association is no longer significant after adjusting for weight loss. On the contrary, lower BMI at-diagnosis and pre-disease BMI are both associated with increased mortality in early-stage upper-GI cancers, compared to overweight/obese individuals [11]. In this ongoing debate, a recent meta-analysis including 45 studies encompassing 607,266 patients with colorectal cancer showed that both obesity and underweight are associated with increased cancer-specific mortality, whereas obesity was also associated with increased odds of overall mortality [10]. However, neither obesity nor overweight was associated with decreased overall survival compared to normal BMI, whereas underweight BMI was associated with decreased odds of overall survival compared to normal BMI; cancer-specific survival could not be studied [10]. Our findings strongly support the concept of the “lung cancer paradox” for the positive impact of overweight and obesity on the long-term outcome of the subset of patients with resectable lung cancer.

### Strengths and Limitations of the Study

The patient population in our study included an extremely representative nationwide sample of consecutive patients. Because of the construction of the prospective database, the proportion of cases lacking data is modest and is not likely to affect results. On the other hand, most of the generally admitted confounding data concerning both patient and tumor-related factors were available, allowing correction for possible confounders. Anyway, some potentially useful parameters are not available: in particular, there is no item concerning social-economic status, muscular burden, of oncogenic driver mutations. Although information on vital status was available for almost all the patients included, long-term cancer-specific events were not available, precluding assessment of relapse-free survival. Finally, information was available on pre-surgery BMI, but not pre-disease BMI, raising the question that underweight could be the loss of weight related to a disease that was diagnosed at an advanced stage more than a patient characteristic independent from the consequence of malignancy.

This study does not answer this question; however, a previous mono-institutional series provided evidence that higher pre-surgical BMI had a protective impact on long-term survival, and showed that pre-disease BMI was also associated with prognosis (worse when underweight, better when overweight and obese), even though weight loss (observed in as many as 45% of patients) was also a predictor of worse outcome [27].

Surgical patients frequently have a variety of comorbidities, such as diabetes, hypertension, coronary artery disease, and chronic obstructive pulmonary disease that could have adverse effects on survival. PS and CCI were also predictors of outcome in our study, independently from BMI. We found that abnormal BMI (in terms of overweight and obesity) was associated with higher CCI. Interestingly, when stratifying BMI for CCI, the detrimental effect of underweight and the protective effect of obesity seemed more pronounced not only in low-comorbidity patients but also in subjects with CCI > 6.

The correlations found between BMI and the different factors studied underlines the complex interplay between body composition and other host-related and tumor-related factors and might provide some insights in understanding mechanisms that explain the impact of underweight on the one side and overweight and, to a greater extent, obesity, on the other.

Underweight is probably an extremely powerful marker of patient frailty. Frailty could be assessed by several other measurements, such as walking speed or walking activity, but morphomics offers the advantage of objective assessment, which is independent of the conditions of measure [28]. In a previous pilot study in patients undergoing pneumonectomy for lung cancer, we showed that underweight was associated with higher C-Reactive Protein (CRP) levels [13]; we also found that systemic inflammation is associated with a lower plasmatic concentration of both albumin and prealbumin [29]. Systemic inflammation and poor nutritional status are related to the scarce infiltration of the tumor by immune cells, which have an established protective impact on resected lung cancer [28]. In particular, we showed that BMI was directly correlated with the number of intratumoral mature dendritic cells, whose number is a positive prognostic factor independent of tumor stage [30].

Inflammatory status may be preexistent or concomitant with lung cancer and is responsible for increased energy consumption, contributing to malnutrition and, in turn, catabolic processes. Similarly, reduced caloric intake, resulting from the malignancy, symptoms, and treatments, may be responsible for fat and muscle loss [29,30]. In a previous mono-institutional series, we showed that sarcopenia (responsible for physical decline, loss of quality of life, and shorter survival) was a frequent feature in underweight patients, but rare in overweight or obese patients with resectable lung cancer [13,14,15,16,17,18,19,20,21,22,23,24,25,26,27]. This observation is consistent with the findings of Prado et al. who showed that sarcopenic obesity is uncommon in lung cancer [31]. Contrary to in underweight patients, it is tempting to speculate that high fat may promote a good state of immune defense against NSCLC: the density of tumor-infiltrating immune cells, in particular mature dendritic cells and cytotoxic CD8+ lymphocytes, is directly correlated with good nutritional status [30].

From a metabolic point of view, the catabolic state of the host, frequently in underweight individuals but probably less frequently in overweight and obese individuals, may be promoted by lack of body reserve, resulting in the enhancement of liver gluconeogenesis and subsequent glucose availability for tumor consumption [32]. On the other hand, glycerol derived from lipolysis sustains gluconeogenesis, a process sparing and delaying the consumption of amino-acids derived from proteolysis [32], suggesting that the availability of fat storage could spare proteins with subsequent attenuation in the loss of skeletal muscle, whereas low-fat reserves force cancer cells to consume more and more amino-acids in a vicious cycle promoting sarcopenia, weight loss, and altered immune response [32].

## 5. Conclusions

In perspective, we could suggest that morphomics and, in particular, BMI, should be integrated into the prognostic evaluation of patients with lung cancer requiring surgery, with the notion that host-related factors are at least as important as tumor-related factors in determining the outcome.

## Figures and Tables

**Table 1:** Characteristics of patients at the time of surgery.

Features	No. Missing (%)	Whole Sample *n* = 54,631	Underweight *n* = 2468	Normal Weight *n* = 25,482	Overweight *n* = 18,765	Obesity *n* = 7877	*p*
Period, no. (%)	0 (0)						<0.0001
2003–2006		7815 (14.3)	357 (14.5)	3888 (15.3)	2657 (14.2)	907 (11.5)	
2007–2010		13,410 (24.5)	581 (23.5)	6342 (24.9)	4632 (24.7)	1844 (23.4)	
2011–2014		16,851 (30.8)	756 (30.6)	7791 (30.6)	5793 (30.9)	2491 (31.6)	
2015–2017		16,555 (30.3)	774 (31.4)	7461 (29.3)	5683 (30.3)	2635 (33.5)	
Sex, no. (%)	1 (<0.1)						<0.0001
Female		16,448 (30.1)	1298 (52.6)	8787 (34.5)	4162 (22.2)	2187 (27.8)	
Male		38,182 (69.9)	1170 (47.4)	16,695 (65.5)	14,602 (77.8)	5690 (72.2)	
Age, mean (SD) y	51 (0.1)	64.1 (10.1)	60.1 (10.5)	63.0 (10.5)	65.6 (9.5)	65.3 (9.1)	<0.0001
Weight, mean (SD) kg	39 (<0.1)	72.9 (15.3)	48.5 (5.7)	64.0 (8.6)	78.9 (8.5)	95.3 (12.7)	<0.0001
Height, mean (SD) cm	39 (<0.1)	169.5 (8.4)	167.3 (8.5)	169.2 (8.4)	170.4 (8.1)	169.2 (8.6)	<0.0001
Body mass index, mean (SD) kg/m^2^	39 (<0.1)	25.3 (4.6)	17.3 (1.0)	22.3 (1.8)	27.1 (1.4)	33.2 (3.3)	<0.0001
Charlson comorbidity index, mean (SD)	0 (0.0)	4.4 (2.2)	3.9 (2.0)	4.2 (2.2)	4.6 (2.2)	4.6 (2.1)	<0.0001
Performance status, no. (%)	2953 (5.4)						<0.0001
0		23,479 (45.4)	845 (36.9)	11,689 (48.4)	8227 (46.4)	2704 (36.3)	
1		23,318 (45.1)	1099 (48.0)	10,301 (42.6)	8047 (45.4)	3861 (51.8)	
2–4		4881 (9.4)	346 (15.1)	2183 (9.0)	1453 (8.2)	892 (12.0)	
Histology, no. (%)	3500 (6.4)						<0.0001
Adenocarcinoma		30,187 (59.0)	1426 (61.2)	14,561 (61.0)	10,181 (58.0)	3998 (54.4)	
Squamous-cell carcinoma		14,176 (27.7)	580 (24.9)	6204 (26.0)	5106 (29.1)	2276 (31.0)	
Carcinoid tumor, typical		1744 (3.4)	54 (2.3)	683 (2.9)	594 (3.4)	412 (5.6)	
Large-cell carcinoma, undifferentiated		1598 (3.1)	96 (4.1)	836 (3.5)	487 (2.8)	178 (2.4)	
Large-cell carcinoma, neuroendocrine		961 (1.9)	51 (2.2)	455 (1.9)	316 (1.8)	139 (1.9)	
Carcinoid tumor, atypical		609 (1.2)	23 (1.0)	232 (1.0)	236 (1.3)	118 (1.6)	
Small-cell lung carcinoma		474 (0.9)	18 (0.8)	212 (0.9)	175 (1.0)	68 (0.9)	
Sarcomatoid carcinoma		385 (0.8)	32 (1.4)	210 (0.9)	101 (0.6)	41 (0.6)	
Other		997 (1.9)	50 (2.1)	474 (2.0)	354 (2.0)	116 (1.6)	
Stage	14,887 (27.3)						<0.0001
0/occult		186 (0.5)	11 (0.6)	96 (0.5)	58 (0.4)	19 (0.3)	
I		21,374 (53.8)	952 (52.8)	9776 (52.6)	7413 (54.3)	3222 (56.7)	
II		7391 (18.6)	352 (19.5)	3531 (19.0)	2518 (18.4)	985 (17.3)	
III		8454 (21.3)	341 (18.9)	3942 (21.2)	2950 (21.6)	1215 (21.4)	
IV		2339 (5.9)	147 (8.2)	1237 (6.7)	709 (5.2)	245 (4.3)	
Surgical procedure, no. (%)	0 (0)						<0.0001
Pneumonectomy		5516 (10.1)	236 (9.6)	2738 (10.7)	1878 (10.0)	662 (8.4)	
Other		49,115 (89.9)	2232 (90.4)	22,744 (89.3)	16,887 (90.0)	7215 (91.6)	
Side, no. (%)	305 (0.6)						0.071
Right		31,412 (57.8)	1460 (59.3)	14,721 (58.1)	10,760 (57.7)	4447 (56.8)	
Left		22,914 (42.2)	1000 (40.7)	10,609 (41.9)	7904 (42.3)	3386 (43.2)	

**Table 2:** Association of body mass index categories with survival. Results presented are pooled hazard ratios over the imputed datasets with 95% confidence intervals.

Features	Unadjusted	Model 1	Model 2	Model 3 *
	*n* = 54,631	*n* = 54,631	*n* = 54,631	*n* = 54,376
	Deaths = 17,094	Deaths = 17,094	Deaths = 17,094	Deaths = 17,043
BMI category				
Underweight	1.24 (1.16–1.33)	1.61 (1.50–1.72)	1.50 (1.40–1.61)	1.51 (1.41–1.63)
Normal weight	1 (reference)	1 (reference)	1 (reference)	1 (reference)
Overweight	0.95 (0.92–0.98)	0.81 (0.79–0.84)	0.83 (0.80–0.86)	0.84 (0.81–0.87)
Obesity	0.88 (0.84–0.92)	0.79 (0.76–0.83)	0.79 (0.75–0.83)	0.80 (0.76–0.84)
Period				
2003–2006	—	1 (reference)	1 (reference)	1 (reference)
2007–2010	—	0.92 (0.88–0.97)	0.93 (0.89–0.98)	0.94 (0.90–0.99)
2011–2014	—	0.93 (0.89–0.97)	0.96 (0.92–1.01)	0.98 (0.94–1.03)
2015–2017	—	1.00 (0.95–1.04)	1.05 (1.00–1.10)	1.08 (1.03–1.14)
Sex, no. (%)				
Female	—	1 (reference)	1 (reference)	1 (reference)
Male	—	3.07 (2.93–3.22)	2.90 (2.77–3.04)	2.90 (2.77–3.04)
Age (per decade)	—	1.08 (1.05–1.10)	1.06 (1.04–1.09)	1.09 (1.07–1.11)
Charlson comorbidity index (per point until a score of 6 **)	—	1.11 (1.10–1.13)	1.09 (1.07–1.10)	1.09 (1.07–1.10)
Performance status				
0	—	—	1 (reference)	1 (reference)
1	—	—	1.22 (1.18–1.27)	1.20 (1.16–1.25)
2–4	—	—	1.67 (1.59–1.76)	1.61 (1.53–1.70)
Surgical procedure				
Pneumonectomy	—	1.75 (1.67–1.83)	1.69 (1.61–1.77)	1.24 (1.18–1.30)
Other	—	1 (reference)	1 (reference)	1 (reference)
Side				
Right	—	1 (reference)	1 (reference)	1 (reference)
Left	—	0.98 (0.95–1.01)	0.98 (0.95–1.01)	0.98 (0.95–1.01)
Histology				
Adenocarcinoma	—	—	1 (reference)	1 (reference)
Squamous-cell carcinoma	—	—	1.04 (1.00–1.08)	1.06 (1.02–1.10)
Carcinoid tumor, typical	—	—	0.29 (0.23–0.37)	0.34 (0.27–0.43)
Large-cell carcinoma, undifferentiated	—	—	1.32 (1.22–1.43)	1.27 (1.18–1.38)
Large-cell carcinoma, neuroendocrine	—	—	1.32 (1.19–1.47)	1.32 (1.19–1.47)
Carcinoid tumor, atypical	—	—	0.54 (0.43–0.67)	0.56 (0.45–0.70)
Small-cell lung carcinoma	—	—	1.66 (1.45–1.90)	1.65 (1.44–1.89)
Sarcomatoid carcinoma	—	—	1.62 (1.38–1.89)	1.57 (1.34–1.83)
Other	—	—	1.18 (1.05–1.32)	1.18 (1.05–1.33)
Stage				
I	—	—	—	1 (reference)
II	—	—	—	1.62 (1.54–1.71)
III	—	—	—	2.33 (2.22–2.44)
IV	—	—	—	2.93 (2.73–3.14)

* Patients with stage 0 or occult were excluded from this model. ** The effect of Charlson comorbidity index was capped at 6: scores above 6 were considered to be associated with the same relative effect as scores of 6.

**Table 3:** Association of body mass index categories with survival stratified by sex. Results are hazard rations with 95% confidence intervals. *p* values compare the hazard ratios (interaction tests).

BMI Category	Females *n* = 16,448 Deaths = 2109	Males *n* = 38,183 Deaths = 14,985	*p*
Unadjusted			
Underweight	1.49 (1.29–1.71)	1.51 (1.40–1.64)	0.83
Normal weight	1 (reference)	1 (reference)	—
Overweight	0.81 (0.72–0.90)	0.85 (0.82–0.88)	0.37
Obesity	0.72 (0.63–0.84)	0.83 (0.79–0.88)	0.069
Model 1 *			
Underweight	1.41 (1.23–1.62)	1.55 (1.43–1.68)	0.25
Normal weight	1 (reference)	1 (reference)	—
Overweight	0.78 (0.70–0.87)	0.83 (0.80–0.86)	0.35
Obesity	0.68 (0.58–0.78)	0.79 (0.75–0.83)	0.049
Model 2 ^†^			
Underweight	1.38 (1.20–1.58)	1.53 (1.42–1.66)	0.19
Normal weight	1 (reference)	1 (reference)	
Overweight	0.81 (0.73–0.91)	0.83 (0.80–0.86)	0.69
Obesity	0.73 (0.63–0.84)	0.80 (0.76–0.84)	0.22
Model 3 ^‡^			
Underweight	1.38 (1.20–1.59)	1.55 (1.42–1.68)	0.18
Normal weight	1 (reference)	1 (reference)	
Overweight	0.82 (0.73–0.91)	0.84 (0.81–0.87)	0.65
Obesity	0.73 (0.63–0.84)	0.81 (0.77–0.86)	0.16

* Adjusted for period, age, CCI, surgical procedure, and side. ^†^ Adjusted for period, age, CCI, surgical procedure, side, performance status, and histology. ^‡^ Adjusted for period, age, CCI, surgical procedure, side, performance status, histology and stage (stage 0 and occult excluded).

## Data Availability

Raw data are available upon reasonable request.

## Supplementary Materials

The following are available online at https://www.mdpi.com/article/10.3390/cancers13184574/s1, Epithor Group: Centers participating in the Epithor Database, Table S1: Overall survival at specific timepoints. Values are percent survival with 95% confidence interval, Table S2: Association of body mass index categories with survival in the original data without imputation. Results pre-sented are hazard ratios with 95% confidence intervals. In these analyses, missing data were not imputed, and only available cases were analysed.
