# Supplementary material for: The Reality of Lung Cancer Paradox: The Impact of Body Mass Index on Long-Term Survival of Resected Lung Cancer. A French Nationwide Analysis from the Epithor Database
Source: Cancers (Basel). 2021 Sep 12;13(18):4574. doi: 10.3390/cancers13184574 (PMC8471205; doi:10.3390/cancers13184574)
Supplement: Supplementary file 1 [file cancers-13-04574-s001.zip › cancers-1340200-supplementary.pdf]

cancers MDPI

Epithor Group: Centers participating in the Epithor database.

Team Leader	Institution
Pascal Thomas	Thoracic Surgery Department – Hopital Nord, Marseille
Brouchet Laurent	Thoracic Surgery Department – Hopital Larrey, Toulouse
Pierre-Emmanuel Falcoz	Thoracic Surgery Department – Hopitaux Universitaires De Strasbourg, Nouvel Hopital Civil
Francoise Le Pimpec-Barthes	Thoracic Surgery Department – Hopital Europeen Georges Pompidou
Eric Steinmetz	Thoracic Surgery Department – CHU Bocage Central
Jacques Jougon	Thoracic Surgery Department – Hopital Cardiologique Du Haut Leveque CHU De Bordeaux
Marc Filaire	Thoracic Surgery Department – Centre Jean Perrin
Valentine Anne	Thoracic Surgery Department – Hopitaux Prives De Metz, Site Robert Schuman
Jerome Mouroux	Thoracic Surgery Department – Hopital Pasteur
Joelle Siat	Thoracic Surgery Department – Hopital De Brabois, CRHU De Nancy
Christophe Peillon	Thoracic Surgery Department – Hopital Charles Nicolle
Serge Haupert	Thoracic Surgery Department – Hopital Henri Duffaut, Centre Hospitalier De La Durance
Michel Aupart	Thoracic Surgery Department – Hopital Trousseau
Antoine Dujon	Thoracic Surgery Department - Clinique Du Cedre
Pierre Alric	Thoracic Surgery Department – Hopital Arnaud De Villeneuve
Gil Frey	Thoracic Surgery Department – Centre Hospitalier Universitaire Grenoble Alpes
Eric De La Roche	Thoracic Surgery Department – Infermiere Protestante
Philippe Boitet	Thoracic Surgery Department - Hopital Privé De L' Estuaire
Nicolas Meneveau	Thoracic Surgery Department – CHU Jean Minjoz
Axel Aubert	Thoracic Surgery Department – Linique Belledonne
Jean Philippe Arigon	Thoracic Surgery Department – Clinique Saint Augustin
Christian Dromer	Thoracic Surgery Department – Polyclinique Bordeaux Nord-Aquitaine
Pierre Corbi	Thoracic Surgery Department – CHU De Poitiers – Site De La Milétrie
Michel Alauzen	Thoracic Surgery Department – Clinique Du Millenaire
Eric Mensier	Thoracic Surgery Department – Hopital Prive La Louvière
Dominique Gossot	Thoracic Surgery Department – Institute Mutualiste Mountsouris
Lotfi Benhamed	Thoracic Surgery Department – Centre Hospitalier De Valenciennes
Marco Alifano	Thoracic Surgery Department – Hopital Cochin, Hopitaux Universitaires Paris Centre
Sebastien Franco	Thoracic Surgery Department – Centre Hospitalier Saint-Louis
Francis Faroy	Thoracic Surgery Department – Centre Hospitalier Privé Saint-Martin
Jalal Assouad	Thoracic Surgery Department – Hopital Tenon, Hopitaux Universitaires Est Paris
Eric Mensier	Thoracic Surgery Department – Polyclinique Du Bois
Gilles Grosdidier	Thoracic Surgery Department – Polyclinique De Gentilly
Pascal Berna	Thoracic Surgery Department – CHU Amiens Picardie, Site Sud
Babak Sadeghi-Looyeh	Thoracic Surgery Department – Clinique Du Ter
Jean-Philippe Avaro	Thoracic Surgery Department – Hopital D'instruction Des Armées Sainte- Anne
Olivier Tiffet	Thoracic Surgery Department – Hopital Nord CHU, Saint-Etienne
Eric Cheysson	Thoracic Surgery Department – Centre Hospitalier René Dubos
Elie Fadel	Thoracic Surgery Department – Centre Chirurgical Marie Lannelongue
Pierre Fourquier	Thoracic Surgery Department – Hospital Privé Du Confluent
Florence Mazeres	Thoracic Surgery Department – Centre Hospitalier De La Cote Basque
Frederique Clerc	Thoracic Surgery Department – Clinique Belharra
Alain Chapelier	Thoracic Surgery Department – Hopital Foch
Luciano Eraldi	Thoracic Surgery Department – Clinique Ambroise Paré
Vincent Blin	Thoracic Surgery Department – Hopital Privé Océane
Marion Durand	Thoracic Surgery Department – Hopital Privé d'Antony
Bernard Lenot	Thoracic Surgery Department – Centre Hospitalier Yves Le Foll
Cedric Perrotin	Thoracic Surgery Department – Clinique Saint-George
Fady Francis	Thoracic Surgery Department – Capio, Clinique Des Cédres
Antonio Minniti	Thoracic Surgery Department – Centre Hospitalier De Pau, Hospital Francois Mitterand
Eric Marcadé	Thoracic Surgery Department – Centre Hospitalier Privé Saint- Grégoire
Jean-Louis Mornex	Thoracic Surgery Department - Hospital Cardio-Vasculaire Et Pneumologique Louis Pradel
Gilles Cardot	Thoracic Surgery Department – Hopital Duchenne CHG
Mayeul Tabutin	Thoracic Surgery Department – Centre Léon Bérard
Eric De La Roche	Thoracic Surgery Department – Clinique De La Sauvegarde
Olivier Chataigner	Thoracic Surgery Department – Polyclinique De Courlancy
Vito-Giovanni Ruggeri	Thoracic Surgery Department – Hospital Robert Debré

Florent Charot	Thoracic Surgery Department – Nouvelle Clinique De L'union
Bertrand Richard De Latour	Thoracic Surgery Department – Polyclinique Saint-Laurent
Alain Prat	Thoracic Surgery Department – Hopital Calmette
Patrick Bagan	Thoracic Surgery Department – Centre Hospitalier Victor Dupouy
Xavier Ducrocq	Thoracic Surgery Department – Rhéna, Clinique De Strasbourg
Pierre-Mathieu Bonnet	Thoracic Surgery Department – Hopital Européen
Philippe Rudondy	Thoracic Surgery Department - Hopital Saint-Joseph,
Bastien Orsini	Thoracic Surgery Department – Centre Hospitalier Privé Clairval
Antoine Guerlin	Thoracic Surgery Department – Clinique Esquirol-Saint Hilaire
Jean-Louis Fasquel	Thoracic Surgery Department – Polyclinique Quimper Sud
John-Tarun Mac Bride Windsor	Thoracic Surgery Department – Hopital D'instruction Des Armées Percy
Emmanuel Martinod	Thoracic Surgery Department – Hopital Avicenne
Christophe Lancelin	Thoracic Surgery Department – Clinique Du Grand Large
Philippe Concina	Thoracic Surgery Department – Centre Hospitalier De Perpignan
Bruno Tremblay	Thoracic Surgery Department – Centre Hospitalier De Meaux, GHEF (Grand Hopital Est Francilien)
Alain Brachet	Thoracic Surgery Department – Hopital Privé Saint-Marie
Pierre Riera	Thoracic Surgery Department – Centre Hospitalier Du Pays D'Aix
Christophe Berton	Thoracic Surgery Department – Pole Santé Oréliance
Arnaud Piquard	Thoracic Surgery Department – CHR d'Orléans
Joaquin Dominguez	Thoracic Surgery Department – Centre Cardiolohique De Nord
Philippe Lacroix	Thoracic Surgery Department – CHU Dupuytren
Eric Brechet	Thoracic Surgery Department – Polyclinique De Limoges-Site Emailleurs Colombier
Patrick Bagan	Thoracic Surgery Department – Centre Hospitalier Victor Dupoy
Philippe Keller	Thoracic Surgery Department – Centre Hospitalier Louis Pasteur
Philippe Dalous	Thoracic Surgery Department – Clinique D'occitanie
Eric Picard	Thoracic Surgery Department – Centre Hospitalier Universitaire Caremeau
Hélène Loubière	Thoracic Surgery Department – Centre Hospitalier Du Mans
Salam Abou Taam	Thoracic Surgery Department – Hopital Privé Claude Galien ICPS
Yves Castier	Thoracic Surgery Department – Groupe Hospitalier Bichat-Claude Bernard
Gerard Pavy	Thoracic Surgery Department – Hopital Privé Arras Les Bonnettes
Olivier Tiffet	Thoracic Surgery Department – Hopital Nord CHU
Xavier De Kerangal	Thoracic Surgery Department – Groupe Hospitalier Du Havre- Hopital Jacques Monod
Olivier Chataigner	Thoracic Surgery Department – Clinique Victor Pauchet De Butler
René Jancovic	Thoracic Surgery Department – Clinique Chirurgicale Du Val d'Or
Christophe Baufreton	Thoracic Surgery Department – Centre Hospitalier Universitaire, Angers
Nicolas Chavanis	Cardiac Surgery Department – Centre Hospitalier Annecy Genevois
Van-Manh Nguyen	Thoracic Surgery Department – Centre Hospitalier Pierre Bérégovoy

Table S1. Overall survival at specific timepoints. Values are percent survival with 95% confidence interval.

Features	1 month	3 months	1 year	3 years	5 years
Overall cohort	97.4 (97.2–97.5)	95.3 (95.1–95.4)	87.2 (86.9–87.5)	69.5 (69.1–69.9)	58.4 (57.9–58.9)
BMI category					
Underweight	96.0 (95.3–96.8)	92.5 (91.4–93.6)	81.1 (79.4–82.8)	61.8 (59.6–64.0)	52.0 (49.7–54.4)
Normal weight	97.2 (97.0–97.4)	94.8 (94.6–95.1)	86.2 (85.7–86.6)	68.7 (68.1–69.4)	57.9 (57.2–58.6)
Overweight	97.6 (97.4–97.8)	95.8 (95.5–96.1)	88.4 (88.0–88.9)	70.4 (69.7–71.1)	58.7 (57.8–59.5)
Obesity	97.6 (97.3–98.0)	96.3 (95.8–96.7)	89.7 (89.0–90.4)	72.3 (71.2–73.4)	61.3 (60.0–62.6)
Sex					
Female	98.9 (98.7–99.0)	98.0 (97.8–98.3)	94.6 (94.2–95.0)	86.3 (85.7–86.9)	80.7 (80.0–81.5)
Male	96.7 (96.5–96.9)	94.1 (93.9–94.4)	84.3 (84.0–84.7)	63.2 (62.7–63.8)	50.4 (49.8–51.0)
Age, y					
≤ 55	98.6 (98.3–98.8)	97.2 (96.9–97.6)	90.0 (89.4–90.7)	74.0 (73.0–74.9)	65.7 (64.6–66.8)
56–60	98.4 (98.1–98.7)	96.9 (96.5–97.3)	89.5 (88.8–90.2)	72.3 (71.2–73.3)	61.2 (60.0–62.5)
61–65	97.7 (97.4–98.0)	95.9 (95.5–96.3)	88.1 (87.4–88.8)	70.4 (69.4–71.4)	59.5 (58.4–60.7)
66–70	97.5 (97.2–97.8)	95.2 (94.8–95.6)	86.8 (86.1–87.5)	69.5 (68.5–70.5)	58.5 (57.4–59.7)
71–75	96.7 (96.3–97.1)	93.9 (93.4–94.5)	85.7 (84.9–86.5)	67.4 (66.3–68.6)	54.0 (52.8–55.4)
> 75	94.8 (94.3–95.3)	91.8 (91.1–92.4)	82.2 (81.3–83.1)	62.0 (60.8–63.2)	48.9 (47.6–50.2)
Charlson comorbidity index					
0–2	98.9 (98.7–99.2)	97.9 (97.6–98.2)	91.6 (91.0–92.2)	77.4 (76.4–78.3)	69.4 (68.2–70.5)
3	98.3 (98.0–98.5)	96.8 (96.4–97.1)	89.5 (88.8–90.1)	73.2 (72.2–74.2)	63.1 (62.0–64.2)
4–5	97.2 (97.0–97.4)	94.9 (94.6–95.2)	86.6 (86.1–87.1)	68.4 (67.7–69.1)	57.0 (56.3–57.8)
≥ 6	95.8 (95.5–96.2)	92.9 (92.4–93.4)	83.5 (82.9–84.2)	63.1 (62.2–64.0)	49.5 (48.5–50.6)
Performance status					
0	98.5 (98.4–98.7)	97.2 (97.0–97.5)	91.3 (90.9–91.7)	75.3 (74.6–75.9)	64.7 (64.0–65.5)
1	97.0 (96.8–97.3)	94.7 (94.4–95.0)	85.9 (85.4–86.4)	67.3 (66.6–67.9)	55.8 (55.1–56.6)
2–4	93.7 (93.0–94.4)	89.2 (88.3–90.1)	76.6 (75.4–77.9)	56.4 (54.9–57.9)	44.6 (43.0–46.2)
Stage					
0	99.4 (98.3–100.0)	96.9 (94.3–99.6)	93.6 (89.8–97.5)	80.2 (73.7–87.3)	68.4 (60.0–78.0)
I	98.2 (98.0–98.4)	96.9 (96.7–97.2)	93.0 (92.6–93.4)	80.1 (79.5–80.7)	68.9 (68.1–69.6)
II	97.3 (96.9–97.6)	94.9 (94.3–95.4)	85.6 (84.7–86.4)	65.5 (64.3–66.7)	53.6 (52.2–55.0)
III	96.3 (95.9–96.7)	93.1 (92.6–93.7)	79.4 (78.5–80.3)	53.9 (52.7–55.1)	42.3 (41.1–43.6)
IV	96.8 (96.0–97.5)	92.7 (91.6–93.8)	75.1 (73.3–77.0)	46.7 (44.5–49.0)	36.3 (34.1–38.6)
Surgical procedure					
Pneumonectomy	94.1 (93.4–94.7)	90.0 (89.2–90.8)	75.7 (74.5–76.9)	53.7 (52.3–55.2)	44.0 (42.5–45.5)
Other	97.7 (97.6–97.9)	95.9 (95.7–96.1)	88.6 (88.3–88.9)	71.4 (70.9–71.8)	60.1 (59.6–60.6)
Side					
Right	97.1 (96.9–97.2)	94.9 (94.7–95.2)	87.1 (86.7–87.5)	70.0 (69.4–70.6)	59.3 (58.6–59.9)
Left	97.8 (97.6–98.0)	95.7 (95.4–96.0)	87.3 (86.9–87.8)	68.7 (68.1–69.4)	57.1 (56.4–57.9)

Table S2. Association of body mass index categories with survival in the original data without imputation. Results presented are hazard ratios with 95% confidence intervals. In these analyses, missing data were not imputed, and only available cases were analysed.

**Features**	**Unadjusted** *n* **= 54,592** **Deaths = 17,075**	**Model 1** *n* **= 54,237** **Deaths = 16,984**	**Model 2** *n* **= 48,002** **Deaths = 14,827**	**Model 3 *** *n* **= 37,674** **Deaths = 11,543**
BMI category				
Underweight	1.24 (1.16–1.33)	1.60 (1.49–1.72)	1.49 (1.38–1.61)	1.48 (1.36–1.62)
Normal weight	1 (reference)	1 (reference)	1 (reference)	1 (reference)
Overweight	0.95 (0.92–0.98)	0.81 (0.79–0.84)	0.84 (0.81–0.87)	0.84 (0.81–0.88)
Obesity	0.87 (0.83–0.92)	0.79 (0.75–0.83)	0.80 (0.76–0.84)	0.83 (0.78–0.87)
Period				
2003–2006	—	1 (reference)	1 (reference)	1 (reference)
2007–2010	—	0.92 (0.88–0.97)	0.94 (0.89–0.99)	0.96 (0.90–1.01)
2011–2014	—	0.93 (0.89–0.98)	0.98 (0.93–1.03)	1.01 (0.95–1.07)
2015–2017	—	1.00 (0.95–1.04)	1.08 (1.02–1.14)	1.12 (1.06–1.19)
Sex, no. (%)				
Female	—	1 (reference)	1 (reference)	1 (reference)
Male	—	3.08 (2.94–3.23)	2.78 (2.64–2.92)	2.78 (2.63–2.94)
Age (per decade)	—	1.07 (1.05–1.10)	1.07 (1.04–1.09)	1.10 (1.07–1.12)
Charlson comorbidity index (per point until a score of 6 **)	—	1.12 (1.10–1.13)	1.09 (1.08–1.11)	1.10 (1.08–1.11)
Performance status				
0	—	—	1 (reference)	1 (reference)
1	—	—	1.20 (1.15–1.24)	1.16 (1.12–1.21)
2–4	—	—	1.65 (1.57–1.74)	1.59 (1.49–1.68)
Surgical procedure				
Pneumonectomy	—	1.75 (1.67–1.82)	1.69 (1.61–1.77)	1.27 (1.20–1.34)
Other	—	1 (reference)	1 (reference)	1 (reference)
Side				
Right	—	1 (reference)	1 (reference)	1 (reference)
Left	—	0.98 (0.95–1.01)	0.98 (0.95–1.01)	0.97 (0.94–1.01)
Histology				
Adenocarcinoma	—	—	1 (reference)	1 (reference)
Squamous-cell carcinoma	—	—	1.05 (1.01–1.09)	1.07 (1.03–1.12)
Carcinoid tumour, typical	—	—	0.19 (0.15–0.25)	0.24 (0.18–0.33)
Large-cell carcinoma, undifferentiated	—	—	1.40 (1.29–1.52)	1.25 (1.13–1.37)
Large-cell carcinoma, neuroendocrine	—	—	1.34 (1.21–1.52)	1.32 (1.16–1.49)
Carcinoid tumour, atypical	—	—	0.48 (0.38–0.61)	0.60 (0.46–0.78)
Small-cell lung carcinoma	—	—	1.73 (1.51–1.99)	2.25 (1.73–2.92)
Sarcomatoid carcinoma	—	—	1.70 (1.46–1.99)	1.52 (1.27–1.82)
Other	—	—	1.18 (1.04–1.33)	1.27 (1.10–1.46)
Stage				
I	—	—	—	1 (reference)
II	—	—	—	1.59 (1.51–1.67)
III	—	—	—	2.35 (2.25–2.46)
IV	—	—	—	3.07 (2.88–3.29)

* Patients with stage 0 (*n* = 81) or occult (*n* = 105) were excluded from this model. ** The effect of Charlson comorbidity index was capped at 6: scores above 6 were considered as associated with the same relative effect as scores of 6.
